# Teaching immunity: A blueprint for medical education

**DOI:** 10.1093/immhor/vlag057

**Published:** 2026-10-26

**Authors:** Bonnie N Dittel

**Affiliations:** Versiti Blood Research Institute, Milwaukee, WI, United States

Although immunology continues to increase in complexity, its foundational concepts including inflammation, immune tolerance, innate immunity, and adaptive immunity are well established. Medical educators therefore have an opportunity to integrate these principles into undergraduate medical education within the context of human health and disease. What has been lacking, however, is a consensus regarding the immunologic topics and disease states that should be covered to ensure that all medical students receive appropriate training in immunology. Establishing such guidance has become increasingly important as inflammation and immune processes are recognized as central contributors to nearly every disease.

To address this need, the American Association of Immunologists (AAI) established the Medical Education Immunology Curriculum Task Force to assess the current state of immunology education in U.S. medical schools and develop AAI-endorsed curricular recommendations. A survey of medical educators revealed considerable variation in both the instructional time devoted to immunology and the topics covered. In response, the task force developed a set of foundational topics and learning objectives, accompanied by relevant clinical conditions illustrating each topic. Together, these recommendations provide medical educators with a practical blueprint for delivering comprehensive and clinically relevant immunology education.

The task force’s findings and recommendations are presented in this issue of *ImmunoHorizons*. Following editorial review, the manuscript was presented to the AAI Council, which unanimously endorsed its recommendations. This work complements a previously published *ImmunoHorizons* article addressing undergraduate immunology education,^1^ thereby extending the journal’s commitment to education across the continuum of immunology training. By publishing authoritative, accessible, and community-informed educational resources, *ImmunoHorizons* is positioned to become an essential resource for immunology educators and to help shape the preparation of the next generation of physicians and scientists.
